# Effect of Thermal Treatment on the Extraction and Antioxidant and Antiglycation Activities of (Poly)phenols from *Ribes magellanicum*

**DOI:** 10.3390/molecules30020318

**Published:** 2025-01-15

**Authors:** Felipe Ávila, Natalia Martinez, Nicolás Mora, Katherine Márquez, Felipe Jiménez-Aspee

**Affiliations:** 1 Department of Nutrition and Food Science, School of Nutrition and Dietetics, Health Science Faculty, Campus Lircay, University of Talca, Talca 3480094, Chile; nmartinez18@alumnos.utalca.cl (N.M.); nmora18@alumnos.utalca.cl (N.M.); 2 Centro de Estudios en Alimentos Procesados CEAP, Campus Lircay, Talca 3480094, Chile; kmarquez@ceap.cl; 3 Department of Food Biofunctionality (140b), Institute of Nutritional Sciences, University of Hohenheim, D-70599 Stuttgart, Germany

**Keywords:** Chilean native berries, thermal treatment, phenolic compounds, antioxidant activity, Maillard reaction

## Abstract

Phenolic compounds have antiglycation activity, but the changes occurring during thermal treatment (TT) in these activities are not completely understood. The effects of the extraction conditions of (poly)phenols from *Ribes magellanicum* fruits, before and after TT, on their antioxidant and antiglycation effects were assessed. (Poly)phenol-enriched extracts (PEEs) from raw and TT (90 °C, 1 h) *Ribes magellanicum* were extracted using three solvent mixtures (ethanol/water/acetic acid) with increasing water content (0, 24, and 49%) and three solvent-to-solid ratios (5, 10, and 20 mL/g). PEEs of raw samples showed increased values of total (poly)phenols (TPC), TEAC, and FRAP and decreased IC_50_ values of fluorescent advanced glycation end products (AGEs) with increasing water content. An increase in TPC and FRAP values was observed for TT samples, but an increase in the IC_50_ values of fluorescent AGEs for PEEs with increasing water content was observed. Antiglycation activity (IC_50_ raw/IC_50_ TT) depended on the solvent-to-solid ratio and the extracting solvent. HPLC-DAD-MS analysis of raw and TT samples showed degradation of anthocyanins, flavonoid fragmentation, and oxidation as the main changes in the phenolic composition of TT samples. We show that TT affects the (poly)phenolic composition of *R. magellanicum*, producing a decrease in the antiglycation activity when extractions are performed with increasing water content, despite increasing TPC and FRAP activity.

## 1. Introduction

Phenolic compounds are secondary metabolites present in plants that are important for human nutrition and the food industry due to their association with a reduced risk of chronic non-communicable diseases, which are currently the first cause of mortality worldwide [1]. Similarly, the intake of foods with low amounts of advanced glycation end products (AGEs) has been associated with a decrease in several markers linked to the development of numerous chronic diseases [2,3,4]. The production of processed foods with low AGE content represents a challenge for the food industry in the forthcoming years [5].

Phenolic compounds can react with electrophilic compounds derived from reducing sugars or their autoxidative reaction products, such as glyoxal and methylglyoxal [6,7]. These reactions have been mainly observed using pure phenolic compounds and mixtures of (poly)phenolic compounds obtained from raw vegetable foods [8]. However, thermal treatment (TT) of foods is a pivotal process that can alter the chemical composition of (poly)phenolic compounds [9,10] and thus their capacity to inhibit the generation of AGEs.

The effect of TT on the antioxidant activity mediated by (poly)phenolic compounds in fruits and vegetables has been previously assessed, with divergent results reported. After TT, the total antioxidant activities of tomatoes (2, 15, and 30 min at 88 °C) [11], sweet corn (25 min, 115 °C) [12], and carrots (2 min, 70 °C, pressure 400–600 MPa for 15 min) [13] were enhanced, while the antioxidant capacities of soybeans decreased after TT [14]. A blackberry juice formulated with *Rubus adenotrichus*, which was thermally processed through pasteurization (88–93 °C, 40 s), showed a decrease in ellagitannins (lambertianin C and sanguiin H-6), with a concomitant increase in the amounts of monomeric ellagic acid [15]. In the strawberry *Fragaria x ananassa*, the TT (95 °C or 60 °C) resulted in a decrease in total anthocyanins following first-order kinetics [16,17]. Interestingly, the TT (40–80 °C) of maqui berries (*Aristotelia chilensis* [Mol] Stuntz) decreases the antioxidant capacity and (poly)phenolic content, but increases the content of free ellagic acid as a result of the hydrolysis of ellagitannins [18].

Chilean native berries have been widely studied for their high antioxidant capacity, which correlates with their (poly)phenolic content [19]. Strong correlations have been reported between the antiglycation activity of berries with the free radical scavenging activity and total phenolic content [20]. *Ribes magellanicum* is a native currant that has been consumed since pre-Hispanic times [21]. Today, these berries are still consumed raw or processed into liqueurs, as well as thermally processed as jams and pasteurized juices, among others [22]. We have demonstrated that TT of another native currant, *Ribes cucullatum*, leads to the inhibition of AGEs in sarcoplasmic proteins incubated with glyoxal, but also to the generation of quinoproteins that are generated from the reaction between sarcoplasmic proteins and oxidized (poly)phenols [23].

The (poly)phenolic composition of *Ribes magellanicum* has been previously reported, with cyanidin 3-glucoside and hydroxycinnamic acids as their main (poly)phenolic constituents [24,25]. A simulated gastrointestinal digestion of methanolic extracts from *Ribes magellanicum* decreased their antioxidant and (poly)phenolic content [26]. However, the optimization of (poly)phenolic compound extraction after TT of *Ribes magellanicum* has not been previously assessed; nor have their antioxidant and antiglycation activities been evaluated.

In this article, we optimized the extraction of phenolic compounds from both raw and thermally treated *Ribes magellanicum* fruits, using three solvents with varying water content and three different solvent-to-solid ratios. We evaluated the changes in the antioxidant and antiglycation activities induced by the TT of the samples. HPLC-DAD-MS analyses were performed to identify key compositional changes induced by the TT.

## 2. Results

### 2.1. Extraction of (Poly)phenols from Raw and Thermally Treated Ribes magellanicum Berries and Their Effects on Antioxidant and Antiglycation Activities

The effect of the thermal treatment (TT) of *Ribes magellanicum* fruits on the antioxidant and antiglycation activities mediated by their (poly)phenols was assessed. *Ribes magellanicum* fruits, raw or TT at 90 °C, were lyophilized, and subsequently (poly)phenols were extracted using solvent-to-solid ratios of 5, 10, and 20 mL/g and also using three solvent mixtures, with increasing water amounts. Extraction yields are presented in Table S1, and the mean value of moisture for the samples was 30.95 ± 2.36%. The mixture of 99:1 ethanol/acetic acid yielded higher amounts of extract from the TT currants. On the other hand, the mixtures ethanol/water/acetic acid (75:24:1) or ethanol/water/acetic acid (50:49:1) yielded higher extraction rates from the raw berries.

**Figure 1:**
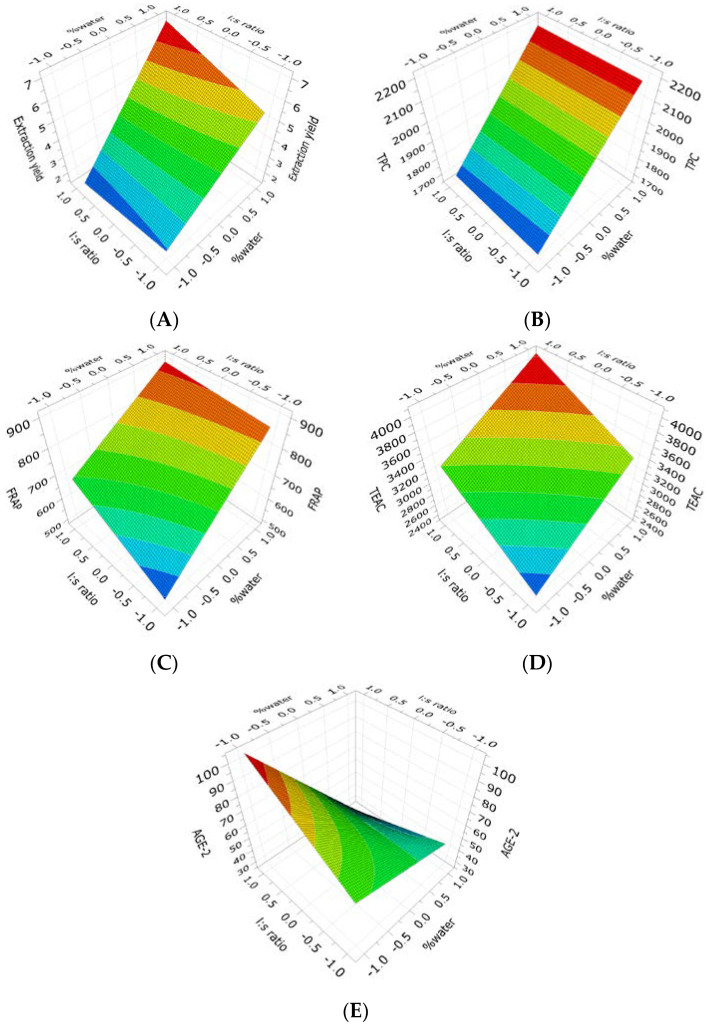
Surface response plot for the effects of the extraction conditions of (poly)phenol-enriched extracts (PEEs) from raw *Ribes magellanicum* fruits using three solvent-to-solid ratios and water content solvents (EtOH/H_2_O/acetic acid: 99:0:1, 75:24:1, and 50:49:1) on the extraction yield, antioxidant activities, and antiglycation activities. (**A**): extraction yield; (**B**): total (poly)phenolic content (TPC); (**C**): FRAP; (**D**): TEAC; and (**E**): AGE-2.

**Figure 2:**
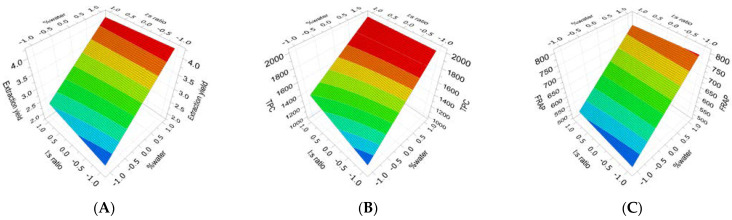

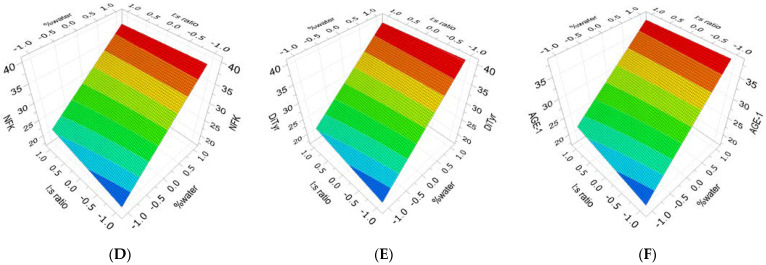
Surface response plot for the effects of the extraction conditions of (poly)phenol-enriched extracts (PEEs) from thermally treated *Ribes magellanicum* fruits using three solvent-to-solid ratios and water content solvents (EtOH/H_2_O/acetic acid: 99:0:1, 75:24:1, and 50:49:1) on the extraction yield, antioxidant activities, and antiglycation activities. (**A**): extraction yield; (**B**): total (poly)phenolic content (TPC); (**C**): FRAP; (**D**): NFK; (**E**): DiTyr; and (**F**): AGE-1.

A two-level full factorial design of experiments (DOE) was performed for raw and TT *Ribes magellanicum* fruits to analyze the effect of the extraction conditions on the extraction yield, as well as on the antioxidant and antiglycation activities. For this, three levels (−1, 0, and +1) and two factors including solvent-to-solid ratios (l:s) and % water were used, and fitted using the partial least squares (PLS) method. The DOE results for raw *Ribes magellanicum* showed a good fit for the extraction yield, TPC, FRAP, TEAC, and AGE-2 responses (Figure 1). On the other hand, the DOE results for TT *Ribes magellanicum* showed a good fit for the extraction yield, TPC, FRAP, NFK, DiTyr, and AGE-1 responses (Figure 2).

The response surface plot depicts a higher extraction yield when 49% water is used, and it was independent of the l:s ratio (Figure 1A). The contribution of this factor is shown in Table 1-Equation (1), indicating that the % water (49%) and the interaction of % water * l:s were proportional to the extraction yield (*p* < 0.05). Figure 1B shows a greater TPC when the percentage of water is 49%, and this parameter was independent of the l:s ratio. The contribution of % water to obtaining a higher TPC is reinforced by the coefficient of the polynomial response model (Table 1-Equation (2)), with only the factor % water being significant and proportional to the TPC. The l:s ratio and their interaction were not significant for TPC. Figure 1C shows higher FRAP values with 49% water and 20 mL/g as the l:s ratio. The contribution of each factor is shown in Table 1-Equation (3). Both factors were significant and proportional to the FRAP value, although their interaction was not significant. Figure 1D shows a greater TEAC capacity, with 49% water and at 20 mL/g as the l:s ratio. The contribution of each factor is shown in Table 1-Equation (4); both factors were significant and proportional to the TEAC value, although their interaction was not significant. Figure 1E shows a lower IC_50_ for AGE-2 in the sample extracted with 49% water (+1) and independent of the l:s ratio. The contribution of this factor is shown in Table 1-Equation (5), with % water and their interaction significant and inversely proportional to the IC_50_ values of AGE-2.

Figure 2 shows the response surface plot for the extraction yield, TPC, and FRAP assay, as well as protein glycation and oxidation induced by (poly)phenols extracted from TT *Ribes magellanicum* at different solvent-to-solid ratios and using a mixture of solvents with increasing amounts of water. The response surface plots showed that the highest extraction yield was reached with 49% water, and independent of the l:s ratio (Figure 2A). The contribution of each factor is shown in Table 1-Equation (6). The parameter % water was significant and proportional to the extraction yield, although their interaction was not significant. Figure 2B shows that the TPC increased with 49% water and was independent of the l:s ratio. The contribution of % water to the increase in the TPC is concomitant with the coefficient of the polynomial response model (Table 1-Equation (7)), which was significant and proportional to the TPC.

Figure 2C shows higher FRAP activity with 49% water, and independent of the l:s ratio. The contribution of each factor is shown in Table 1-Equation (8). The % water was significant and proportional to the FRAP activity, although their interaction was not significant. Figure 2D shows a lower IC_50_ value for NFK with 0% water and independent of the l:s ratio. The contribution of this factor is shown in Table 1-Equation (9). It was found that the parameter % water was significant and proportional to the IC_50_ values of NFK. Figure 2E shows a lower IC_50_ value for DiTyr with 0% water and was independent of the l:s ratio. The contribution of this factor is shown in Table 1-Equation (10). The % water was significant and proportional to the IC_50_ values of DiTyr. Figure 2F shows lower IC_50_ values for AGE-1 when PEEs were extracted with 0% water and independent of the l:s ratio. The contribution of this factor is shown in Table 1-Equation (11). It can be observed that the % water was significant and proportional to the IC_50_ values for AGE-1. These results showed that in TT samples, the variable l:s ratio and their interaction were not significant for TPC, FRAP, yield, NFK, AGE-1, or DiTyr.

**Figure 3:**
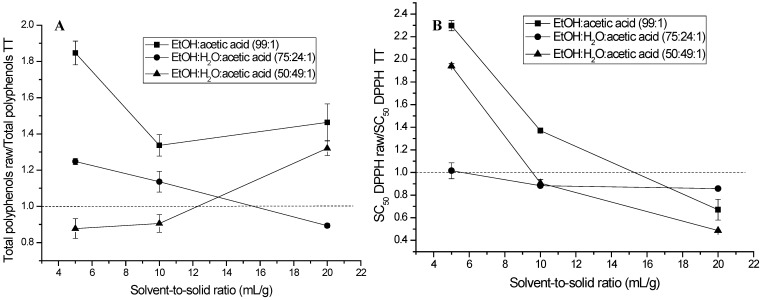

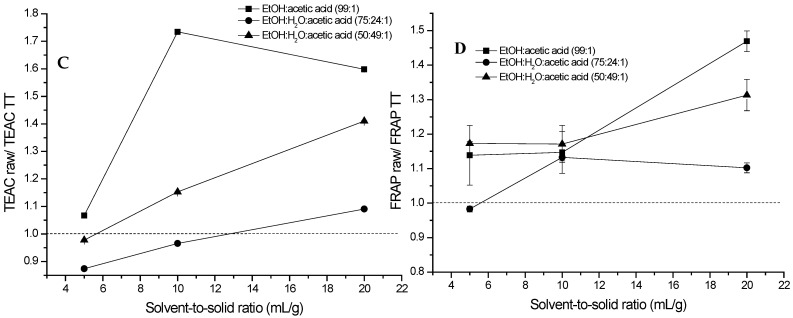
Effect of thermal treatment of *Ribes magellanicum* fruits and the extraction conditions (solvent-to-solid ratios and different solvents) on antioxidant-related activities. Panel (**A**): total (poly)phenolic amount; Panel (**B**): DPPH activities; Panel (**C**): TEAC; and Panel (**D**): FRAP.

Figure 3 shows the effect of the extraction conditions when comparing raw and TT samples on antioxidant activities (DPPH, TEAC, and FRAP) and total (poly)phenol content. Figure 3A shows the ratio between total raw (poly)phenols and total TT (poly)phenols, which depends on the solvent-to-solid ratio but also shows a dependence on the system of solvents used to perform the extractions. The ratio decreased by increasing solvent-to-solid ratios for solvent mixtures with 0% water and 24% water. However, the ratio increased with increasing solvent-to-solid ratios for the mixture of solvents containing 49% water.

Figure 3B shows that the ratio of SC_50_ DPPH raw/SC_50_ DPPH TT decreased when increasing the solvent-to-solid ratio for the three systems of solvents used in this study, with TT samples with a low solvent-to-solid ratio being more efficient than raw samples. Figure 3C,D show an increase in the ratios of TEAC raw/TEAC TT and FRAP raw/FRAP TT(with coefficient raw/TT values > 1.0) when increasing the solvent-to-solid ratio for the three systems of solvents used in this study. In the case of raw TEAC/TT TEAC, the samples at low solvent-to-solid ratios, namely 5 and 10 mL/g for 24% water and 5 mL/g for EtOH/H_2_O/acetic acid (99:1), showed higher efficiency of TT samples over raw samples. For the ratio raw FRAP/TT FRAP, only the sample of 5 mL/g extracted with the system EtOH/H_2_O/acetic acid (75:24:1) showed higher efficiency of TT samples over raw samples.

### 2.2. Extraction of (Poly)phenols from Raw and Thermally Treated Ribes magellanicum and Their Effects on Protein Oxidative Modifications and Advanced Glycation End Products

**Figure 4:**
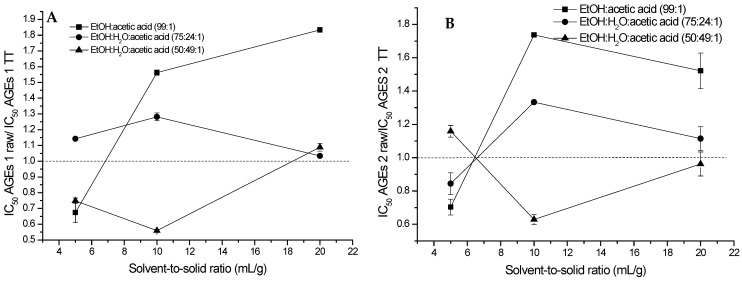

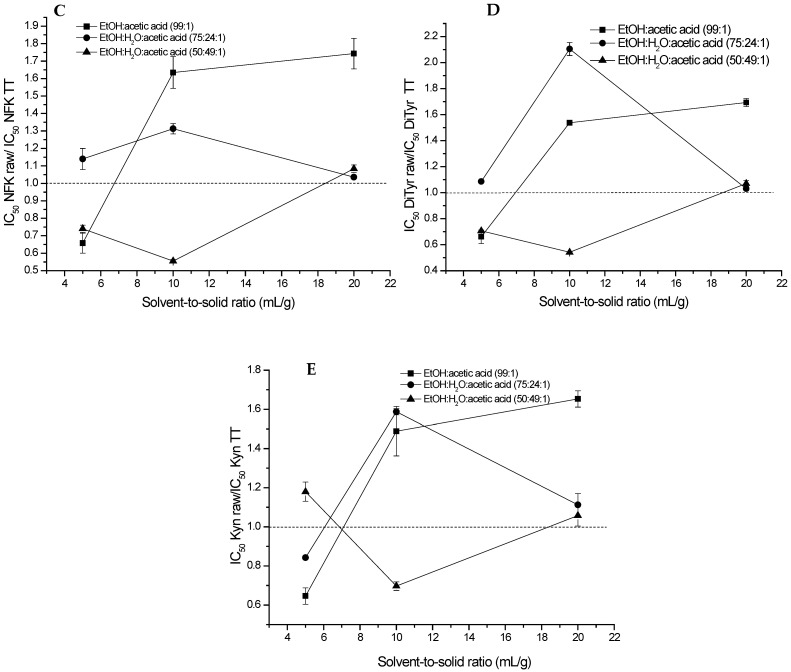
Effect of thermal treatment of *Ribes magellanicum* fruits on the inhibition of protein glycation and oxidative modifications and the solvent-to-solid ratio, using three different solvent systems. Panel (**A**) shows the effect on AGE 1 (λ_EXC =_ 325/λ_EM_ = 440 nm). Panel (**B**) shows the effects on AGE 2 (λ_EXC_ = 389/λ_EM_ = 443 nm). Panel (**C**) shows the effect on n-formylkynurenine (NFK, λ_EXC_ = 325/λ_EM_ = 434 nm). Panel (**D**) shows the effect on dityrosine (DiTyr, λ_EXC_ = 330/λ_EM_ = 415 nm) modifications and Panel (**E**) shows the effects on kynurenine (Kyn, λ_EXC_ = 365/λ_EM_ = 480 nm) modifications.

The effects of the three types of solvent mixtures and solvent-to-solid ratios used for the extraction of (poly)phenols from raw and TT *Ribes magellanicum* on the IC_50_ values of oxidative modifications (N-formyl Kyn, Kyn, and DiTyr) and fluorescent AGEs (AGE 1 and AGE 2) of proteins incubated with glucose are depicted in Figure 4.

The efficiency of PEEs TT in the inhibition of the generation of AGE 1, N-formyl Kyn, and DiTyr increases with higher solvent-to-solid ratios, with the samples at 20 mL/g being more efficient than the TT samples (Table S2), independently of the system of solvent used to perform the extraction (Figure 4A,C,D). Figure 4B,E show a similar trend for 0% water and 24% water, with increased efficiency at inhibiting the generation of Kyn and AGE 2 of TT samples at higher solvent-to-solid ratios.

### 2.3. HPLC-DAD-MS-MS Analysis of the Changes Occurring in PEEs from Raw and Thermally Treated Ribes magellanicum

**Figure 5:**
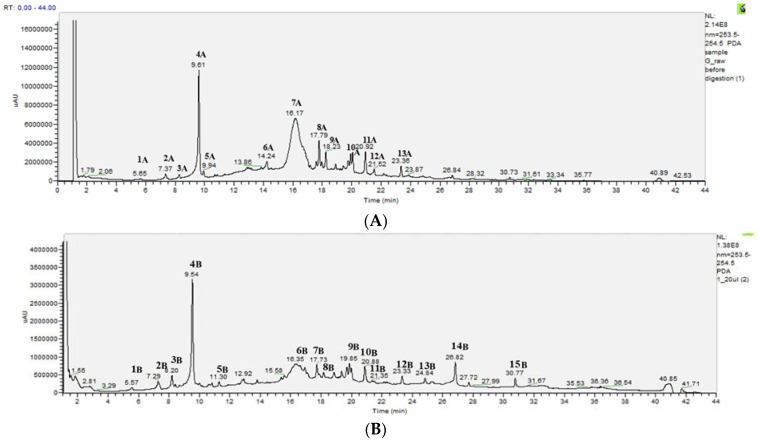
Representative HPLC-DAD chromatograms of (poly)phenol-enriched raw (**A**) and thermally treated (**B**) extracts from *Ribes magellanicum* fruits. Detection: UV, 254 nm.

The chemical composition of PEEs from samples from raw and TT *Ribes magellanicum*, extracted with 24% water at 20 mL/g, was determined by HPLC-DAD-MS-MS, considering that at this proportion there was a high efficiency of TT samples to inhibit AGEs when compared with raw samples. Figure 5A,B show the HPLC-DAD chromatograms (at 254 nm) of the PEEs from raw and TT *Ribes magellanicum* at 20 mL/g, respectively. Twelve main peaks for PEEs from raw samples were observed, whereas in the TT samples a total of 15 main peaks were detected (Figure 5).

Table 2 summarizes the main peaks detected at 254 nm and a tentative identification through mass spectrometry analyses. Table 2 shows that dehydrated and oxidized phenolics were tentatively identified in samples after thermal treatment (peaks 3B, 5B, and 15B). In addition, Table S1 shows the changes in the cyanidin 3-glucoside amounts for raw and thermally treated PEEs from *Ribes magellanicum* fruits in the different extracting conditions used in this study.

## 3. Discussion

It has been proposed that solvent type and solvent-to-solid ratio are some of the main parameters affecting the yield of (poly)phenol extraction [27]. The yield of extraction of (poly)phenols depends, among other factors, on the polarity of the solvent and the phenolic constituents of the sample. The extraction of total (poly)phenols from *Ribes nigrum* L., *Ribes rubrum* L., and *Ribes uva crispa* L. increased when the extraction was carried out with methanol/water (50:50) when compared with only water or only methanol [28]. Furthermore, it has been reported that the maximum extraction yield of anthocyanins from *Ribes nigrum* was reached at 60% ethanol, whereas the experimental concentrations employed were 40, 60, and 96% ethanol [29]. In another study with *Ribes nigrum*, the highest antioxidant activity was observed with the binary solvent mixture EtOH:H_2_O in a ratio of 7:3, in which the experimental ethanol concentrations were from 40 to 80% [30]. On the other hand, the effect of acidification has been previously assessed on strawberries, *Fragaria ananassa* species, and it was shown to be the most efficient at 1% [31]. However, the optimization of the extraction of phenolic compounds from *Ribes magellanicum* has not been previously evaluated, and experiments related to the bioactivity of this fruit have been carried out using the solvent ETOH/acetic acid, 99:1 [24,25]. Consequently, in this work, we selected three solvent mixtures (ethanol/water/acetic acid) at 99, 75, and 50% in ethanol, maintaining the acetic acid amount at a constant 1%. The extraction of raw samples of *Ribes magellanicum* with a higher % water correlated with an increase in the extraction yield, TPC, antioxidant activity, and inhibitory activity of AGE 2. However, when *Ribes magellanicum* is thermally treated, the highest extraction yield, TPC, and FRAP were reached with 49% water (50% EtOH), but higher inhibitory effects in the generation of NFK, AGE 1, and DiTyr, induced by the incubation of glucose with BSA, were observed at 0% water (99% EtOH). Cruz et al. have recently shown that IC_50_ for the inhibition of fluorescent AGEs produced by the glycation of BSA by glucose decreases after thermal treatment under oxidative conditions in secondary metabolite-enriched extracts from *Phaseolus vulgaris* and procyanidin C1 [32]. IC_50_ in fluorescent AGEs mediated by secondary metabolite-enriched extracts from *Phaseolus vulgaris* was 513.8 ± 23.6 μg/mL [32]. In the present article, we show that PEEs from *Ribes magellanicum* extracted using EtOH: H_2_O, 99:1 as a solvent and a solvent-to-solid ratio of 20 mL/g showed an IC_50_ of 48.72 ± 4.22 μg/mL for AGEs1 and 118.91 ± 1.67 μg/mL for AGEs2; this was the least efficient condition assessed in this study (Table S2). On the other hand, it has been shown that water/ethanol extracts (70:30), at 45°C for 90 min, elicited IC_50_ values from 480 to 1200 μg/mL for black chokeberry and strawberry, respectively [33]. Harris et al. have also analyzed the antiglycation activity of different berries, including *Ribes glandulosum* Grauer and *Ribes Triste* Pall, showing IC_50_ values for fluorescent AGEs of 46.0 and 208.6 μg/mL for extracts prepared using 80% ethanol as a solvent [20].

We have recently shown that TT under oxidative conditions of secondary metabolite-enriched extracts from *Phaseolus vulgaris* beans has been shown to increase the antioxidant activity by means of TEAC and FRAP, as well as the inhibition of lipid peroxidation of fatty meats generated after gastric digestion [29]. Oxidation of (poly)phenols such as quercetin, fisetin, and isorhamnetin, among others, has been shown to increase some antioxidant activity indexes [34]. In this study, it is demonstrated that the antioxidant and antiglycation activities of (poly)phenols from *Ribes magellanicum* after TT depends on the extraction conditions of the (poly)phenols.

The (poly)phenolic composition of polyphenol-enriched extracts from raw *Ribes magellanicum* has been previously reported [24,25], and HPLC-DAD-MS analyses of the raw samples shown in this article are consistent with those reports. However, optimization studies assessing extraction conditions and their effects on antioxidant and antiglycation activities mediated by polyphenols from raw and thermally treated *Ribes magellanicum* fruits have not been previously conducted. In this article, we also show a characterization of the main changes in the (poly)phenolic composition by HPLC-DAD-MS analyses. In raw samples, the peaks ranging from 7.37 to 9.61 min correspond to caffeoyl quinic acid derivatives; the fragmentation pattern for these compounds agrees with previous reports [24,35]. For TT samples, the peak at 8.2 min (peak 3) shows a fragmentation pattern that was compatible with dehydrated 3-caffeoyl quinic acid, which agrees with the compounds identified after the degradation of caffeoylquinic acid [35]. The area corresponding to peak 4A (9.64 min) for TT samples decreased by 51% compared with that of peak 4B (9.54 min) in raw samples. For raw samples, the peaks at 9.95 min (peak 5A) and 14.24 min (peak 6A) were tentatively identified as caffeoyl hexoside and feruloyl quinic acid, respectively, which were not present in samples after TT (Figure 5B) and agree with previous results [25]. The peak 7A at 16.17 min for raw samples and the peak 6B at 16.35 min for TT samples were tentatively identified as cyanidin hexoside and cyanidin rhamnoside, respectively. The area of the peak 7A decreased by 81.9% for the PEEs of *Ribes magellanicum* after TT (Figure 5B). This agrees with the decrease in the mean value of cyanidin 3-glucoside for the sample G (ETOH: H_2_O: CH_3_COOH; 75:24:1; solvent-to-solid ratio, 20 mL/g), which showed mean values of 61.4 ± 15.2 μmol/g PEE for raw samples and 10.5 ± 2.2 μmol/g PEE for thermally treated samples (Table S1). The degradation of anthocyanins due to TT has been assessed over recent decades [36,37,38]. The catabolites observed arise from different chemical reactions, including (1) cleavage reactions (including deglycosylation) that yield chalcone intermediates, which further produce phenolic acids [9,17], (2) Baeyer–Villiger oxidation mediated by hydrogen peroxide, generating oxidized intermediates like anthocyanones (8-β-D-glucopyranosyl-2,4-dihydroxy-6-oxo-cyclohexa-2,4-dienyl acetic acid) [39], and (3) free radical reactions during thermal processing that produce a benzofuranone derivative, such as (2-(3,4-Dihydroxyphenyl)-4,6-dihydroxybenzofuran-3-carboxylic acid glucose ester), as a result of the reaction between cyanidin 3-O-glucoside and 2, 20-azobis-(2,4-dimethyl)valeronitrile [40]. Additionally, recent reports indicate that querecetin oxidation generates 2-(3,4-dihydroxybenzoyl)-2,4,6-trihydroxy-3(2H)-benzofuranone [41].

This oxidation product was 200 times more effective than quercetin in inducing cellular cytoprotection and antioxidant capacity in Hs68 and Caco-2 cells [41]. This highlights the relevance of studying oxidative processes that occur during thermal processing. The peak 8A at 17.79 min for raw samples and the peak 7B at 17.73 min were tentatively identified as kaempferol derivatives, with the fragmentation pattern agreeing with a previous report [34]. The peak 8B eluting at 18.2 min was tentatively identified as phloroglucinaldehyde, according to the fragmentation pattern, which was consistent with previous reports which found phloroglucinaldehyde as a breakdown product of anthocyanin degradation [39]. The identification of phloroglucinaldehyde also agreed with pharmacokinetic experiments after the intake of 500 mg of ^13^C-labeled-cyanidin 3-glucoside, which identified phloroglucinaldehyde in urine, serum, and feces [42]. For TT samples, the peak 9B (19.85 min) was tentatively identified as rutin, according to the fragmentation pattern, which agrees with a previous study [43]. For TT samples, the peak 11 eluting at 21.35 min was tentatively identified as kaempferol 3-0-rutinoside, agreeing with earlier reports regarding the precursor ion and the fragmentation pattern [44]; this compound was also detected in raw samples. One of the main differences between raw and TT samples was observed in the retention times corresponding to peaks 14B and 15B, which were not present in raw samples. The compound present in peak 14B was tentatively identified as kaempferol rhamnoside. The peak 15B was tentatively identified as a carboxylated oxidation compound from quercetin based on its [M−H^−^] ion at 347 m/z and its absorption spectra, which showed a loss of 44 amu consistent with the loss of -CO_2_ moiety [45], showing a peak at 303 m/z, followed by a loss of 233 amu. Similar compounds have been reported to be generated from quercetin oxidation mediated by Au (III) in acid conditions, showing methoxylated quinone derivatives of quercetin [46], but not compatible with the fragmentation pattern of the compound detected as peak 15B in this study. It is interesting to note that TT can induce chemical modifications, including (poly)phenol fragmentation and deglycosylation, as shown in this work. However, it is also important to consider that the observed differences between raw and TT samples could be due to changes in the extraction process, due to changes in the polarity of the compounds during TT.

## 4. Materials and Methods

### 4.1. Plant Material

*Ribes magellanicum* fruits were collected in January of the year 2022 at the National Park Conguillio, IX region, Chile (39°29′36″ S; 71°41′53″ W). Samples were transported at 4 °C and then frozen at −20 °C until use.

### 4.2. Thermal Treatment of Ribes magellanicum

*Ribes magellanicum* samples were divided into raw and thermally treated (TT) samples. Raw samples were freeze-dried without thermal treatment (Biobase, model, BK-FD10P, Jinan, China). For the TT, 50 g of samples was defrosted and then exposed to 90 °C for 1 h in a convection oven (ChefTop, XVC705E, UNOX, Cadoneghe, Italy). After TT, samples were weighed and frozen at −20 °C and then freeze-dried until posterior treatment.

### 4.3. Polyphenol-Enriched Extract Preparation

Lyophilized raw and TT samples of *Ribes magellanicum* (aprox.13–14 g) were homogenized in a blender to form a powder. The powder was extracted using three different mixtures of solvents: (1) EtOH/acetic acid (99:1), (2) EtOH/H_2_O/acetic acid (75:24:1), and (3) EtOH/H_2_O/acetic acid (50:49:1). These conditions correspond to the design of experiments (DOE) 3^2^ and the variables given in Table 3.

For each solvent mixture, 3 solvent-to-solid ratios (5, 10, and 20 mL/g) were used to carry out the extraction. Samples were sonicated using a bath sonicator with an ultrasonic power of 120 W for 20 min at 25 °C (Isolab, model 621.05.003, Eschau, Germany). The extraction with the same volume of solvent and posterior sonication were repeated 4 times for each sample. Then, extracts were combined and evaporated under reduced pressure. To obtain the secondary metabolite-enriched extracts, these samples were diluted with milli-Q water and adsorbed into Amberlite XAD-7 (DuPont de Nemours, Inc., Wilmington, DE, USA) (previously activated and washed) in a batch of 200 g. Amberlite XAD-7 allows the removal of sugars, proteins, and inorganic salts. The batch was left under agitation for 30 min at room temperature. Then, the resin was washed with milli-Q water, and the compounds were recovered with acidified ethanol. Then, the extracts were evaporated and finally lyophilized to dryness. Extracts were stored at −20 °C until analysis. Extraction yields were calculated based on dry fruit weight and are shown in Table S1.

### 4.4. Antioxidant Assays

#### 4.4.1. DPPH Scavenging Assay

The free radical scavenging effect of the PEEs from raw and TT *R. magellanicum* was assessed by the reduction of the absorbance of a methanolic solution of DPPH. Reaction mixtures containing test samples (dissolved in MeOH) and 400 µM DPPH ethanolic solutions in 96-well microliter plates were incubated at 37 °C for 30 min, and absorbances were measured at 515 nm (Biotek, ELx800, Shoreline, WA, USA). Gallic acid was used as the positive control and percent inhibition by sample treatment was determined by comparison with a MeOH-treated control group. SC_50_ values denoted the concentration of sample required to scavenge 50% DPPH free radicals. Results are expressed as (µg PEE/L).

#### 4.4.2. TEAC Assay

The ABTS radical cation (ABTS.^+^) assay was used to evaluate the radical scavenging activity of the PEEs from raw and TT *Ribes magellanicum* [47]. Briefly, ABTS.^+^ stock solution was prepared by adding 10 mL of 7 mM 2, 2-azobis (2-amidinopropane) dihydrochloride to 179 mL of 140 mM aqueous potassium persulfate. The mixture was incubated for 12 h at room temperature in the dark. Working ABTS.^+^ solution was prepared by diluting ABTS.^+^ stock solution 20 times with PBS for further use. A total of 700 µL of ABTS.^+^ solution was mixed with 20 µL of the sample, and after incubation for 6 min in the dark, the decrease in absorbance at 734 nm was monitored with a spectrophotometer (Cary 8454, Agilent, Santa Clara, CA, USA). The ABTS radical scavenging activities of the extracts are expressed as mg Trolox equivalents/g PEE.

#### 4.4.3. FRAP Assay

This assay was carried out according to previous reports [48]. Briefly, a FRAP solution composed of 10 mM TPTZ (2,4,6-tri(2-pyridyl)-S-triazine), 300 mM sodium acetate, and 20 mM FeCl_3_ in a ratio of 1:10:1 (v/v/v) was prepared. Samples, blanks, or standards (150 µL) were mixed with 2850 µL of FRAP solution and incubated for 30 min in the dark at room temperature. Changes in absorbance were recorded at 590 nm with a spectrophotometer (Cary 8454, Agilent, Santa Clara, CA, USA). FRAP values were expressed in µmol Trolox equivalents (TE)/g of raw or TT PEE samples.

### 4.5. Determination of Total Phenolic Content (TPC)

Analysis of the TPC of PEEs was carried out following the Folin–Ciocalteu method, as described previously [49]. Briefly, the (poly)phenol extract was resuspended at 1 mg/mL, and an aliquot of 30 μL was mixed with 10% Folin–Ciocalteu reagent (*w*/*v*), followed by an aliquot of 240 μL of 5.0% Na_2_CO_3_ (*w*/*v*). The mix was incubated at 37 °C in the dark for 30 min, and then absorbance was measured at 765 nm in a microplate reader (Biotek, ELx800, USA). A calibration curve of gallic acid was used to estimate the equivalents of gallic acid (GAEs) present in the sample.

### 4.6. Inhibition of Fluorescent AGEs, Kynurenine and Dityrosine Generation, and Tryptophan Consumption

BSA (10 mg/mL) was incubated with glucose (0.5 M) and sodium azide (0.2 mg/mL) for 40 h at 55 °C, in the presence or absence of different concentrations of PEEs (1 to 500 μg/mL) or aminoguanidine (1 to 500 μg/mL) as a reference compound prepared in phosphate buffer (100 mM, pH 7.4), as previously reported [6]. Samples and controls were diluted by taking 0.5 mL of samples or controls to 3 mL with phosphate buffer (100 mM, pH 7.4) and measuring the fluorescence (Perkin Elmer LS 55 spectrofluorimeter, Perkin Elmer Ltd. Waltham, MA, USA). The fluorescence values of AGEs1, AGEs2, kynurenine, and dityrosine were quantified, and determined at λ_EXC_/λ_EM_ = 325/440 nm, λ_EXC_/λ_EM_ = 389/443 nm, λ_EXC_/λ_EM_ = 365/480 nm, and λ_EXC_/λ_EM_ = 330/415 nm, respectively. The inhibition percentage of AGEs, Kyn, or Trp was calculated at different extract concentrations, according to Equation (12):

% Inhibition = (1 − (BSAGlu/P − BSAP)/BSAGlu) × 100(12)

where BSAGlu/P = fluorescence intensity of serum albumin incubated with glucose and PEEs, standard compounds, or aminoguanidine; BSAP = fluorescence intensity of serum albumin incubated with PEEs, standard compounds, or aminoguanidine at the same concentration as BSAGlu/P, and BSAGlu = fluorescence intensity of serum albumin incubated with glucose. Values were expressed as the half maximal inhibitory concentration (IC_50_) in μg/mL.

### 4.7. Determination of Phenolic Compounds by HPLC-DAD-MS-MS

Chromatographic separation of the main (poly)phenolic compounds was performed on an HPLC (Shimadzu, Kyoto, Japan) consisting of an LC-20AT pump, an RF-20A multi-wavelength detector, a SPD-M20A UV diode array detector, a DGU-20A3r degasser, an SIL-20AC-HT autosampler, and a CBM-20A system controller. Instrumental control and analysis of data were performed with LabSolutions software (version 5.96), (Shimadzu, Duisburg, Germany). A Kinetex PFP column was used (250 × 4.6 mm, 5 µm, Phenomenex) at a flow rate of 0.8 mL/min using a linear gradient solvent system which is based on H_2_O/formic acid/ACN (87:5:3, v/v/v, solvents A) and H_2_O/formic acid/ACN (40:5:50, v/v/v, solvents B). The initial conditions were 95% A and 5% B. The gradient started with 95% A and 5% B. The ratio of solvents was then changed to 75:25 A:B in 50 min and returned to initial conditions (95:5 A:B) at 55 min. The system was left at the initial proportion of solvents for 10 min more before the next injection. The volume injected was 20 µL. The phenolic compounds were quantified by external calibration at the maximum wavelength of each family of compounds (520 nm for anthocyanins, 320 nm for hydroxycinnamic acids, and 360 nm for flavonols) using cyanidin-3-glucoside, chlorogenic acid, and quercetin-3-glucuronide as standards, respectively according to Jiménez-Aspee et al. [24].

### 4.8. Statistical Analysis

Statistical differences between the mean value between samples and controls were determined by one-way analysis of variance (ANOVA) followed by Tukey post hoc test using the software SPSS14.0 for Windows (IBM, Armonk, NY, USA), and the significance was set at *p* < 0.05. For this study, relevant factors contributing to each extract response (TPC, DPPH, FRAP, TEAC, extraction yield, NFK, AGE-1, DiTyr, Kyn, and AGE-2) were identified. The design of experiments (DOE) was performed using Modde v.7.0 software (Umetrics, Umeå, Sweden). The factorial DOE was developed with two independent variables and three levels (3^2^); three replicates were considered, along with the partial least square (PLS) algorithm used for model fitting. The description of the DOE 3^2^ is shown in Table S2 in the Supplementary Materials. As a criterion of the goodness of fit, the percentage of variation in the response explained by the model (R^2^ > 0.50) was considered, according to previous studies [50].

## 5. Conclusions

The effect of the extraction conditions on the yield, antioxidant, and antiglycation activities of (poly)phenols from *Ribes magellanicum* before and after TT was assessed for the first time. Raw samples of *Ribes magellanicum* showed an increase in extraction yield, TPC, FRAP, and TEAC assays with a higher water content. This trend agrees with the increase in the inhibitory activity of AGE 2 by PEEs when the extraction was carried out at higher water amounts. However, after TT an increase in TPC and FRAP values was observed, but a change in the trend was observed for the IC_50_ values of fluorescent AGEs, which decreased their efficiency at higher water amounts. Antiglycation activity (IC_50_ raw/IC_50_TT) strongly depended on the solvent-to-solid ratio and the extracting solvent. HPLC-DAD-MS analysis of raw and TT samples showed degradation of anthocyanins, flavonoid fragmentation, and oxidation as the main changes in the phenolic composition of TT samples. We show for the first time that the extraction conditions of *Ribes magellanicum* are a key factor associated with the antiglycation activity of phenolic compounds after TT.

## Figures and Tables

**Table 1:** Partial least square (PLS) summary model and polynomial coefficients for responses associated with extraction yield, antioxidant activity, and protein glycation and oxidation inhibition mediated by polyphenol-enriched extracts from raw and thermally treated *Ribes magellanicum* fruits.

Sample	Response	PLS Model	Coefficients
Raw Ribes	Yield	R^2^ = 0.887; Q^2^ = 0.598 RSD = 0.714 Reproducibility = 0.98	3.95 Cte. + 1.96 × X_1_ + 0.23 × X_2_ (ns) + 0.40 × [X_1_ × X_2_] (Equation (1))
Raw Ribes	TPC	R^2^ = 0.809; Q^2^ = 0.447 RSD = 111 Reproducibility = 0.93	1938 Cte. + 214 × X_1_ + 7.2 × X_2_ (ns) − 2.6 × [X_1_ × X_2_] (ns) (Equation (2))
Raw Ribes	FRAP	R^2^ = 0.767; Q^2^ = 0.318 RSD = 81 Reproducibility = 0.93	737 Cte. + 126 × X_1_ + 48.9 × X_2_ − 28.3 × [X_1_ × X_2_] (ns) (Equation (3))
Raw Ribes	TEAC	R^2^ = 0.840; Q^2^ = 0.453 RSD = 239 Reproducibility = 0.98	3295 Cte. + 400 × X_1_+ 346 × X_2_ − 46.8 × [X_1_ × X_2_] (ns) (Equation (4))
Raw Ribes	AGE-2	R^2^ = 0.654; Q2 = 0.225 RSD = 18 Reproducibility = 0.98	59.6 Cte. − 20.5 × X_1_ + 4.62 × X_2_ (ns) − 11.1 × [X_1_ × X_2_] (Equation (5))
TT Ribes	Yield	R^2^ = 0.554; Q^2^ = 0.306 RSD = 0.744 Reproducibility = 0.97	3.20 Cte. + 0.87 × X_1_ + 0.101 × X_2_ (ns) − 0.112 × [X_1_ × X_2_] (ns) (Equation (6))
TT Ribes	TPC	R^2^ = 0.647; Q^2^ = 0.329 RSD = 282 Reproducibility = 0.99	1614 Cte. + 344 × X_1_ + 81.6 × X_2_ (ns) − 68.1 × [X_1_ × X_2_] (ns) (Equation (7))
TT Ribes	FRAP	R^2^ = 0.581; Q^2^ = 0.281 RSD = 104 Reproducibility = 0.94	639 Cte. + 121 × X_1_ + 5.77 × X_2_ (ns) − 10.9 × [X_1_ × X_2_] (ns) (Equation (8))
TT Ribes	NFK	R^2^ = 0.595; Q^2^ = 0.561 RSD = 6.95 Reproducibility = 0.99	29.3 Cte. + 8.28 × X_1_ + 1.02 × X_2_ (ns) − 1.10 × [X_1_ × X_2_] (ns) (Equation (9))
TT Ribes	DiTyr	R^2^ = 0.589; Q^2^ = 0.540 RSD = 8.02 Reproducibility = 0.99	29.9 Cte. + 8.89 × X_1_ + 0.99 × X_2_ (ns) − 1.12 × [X_1_ × X_2_] (ns) (Equation (10))
TT Ribes	AGE-1	R^2^ = 0.590; Q^2^ = 0.552 RSD = 6.80 Reproducibility = 0.98	29.0 Cte. + 7.84 × X_1_ + 1.11 × X_2_ (ns) − 1.12 × [X_1_ × X_2_] (ns) (Equation (11))

RSD = residual standard deviation; Cte = constant factor; X_1_ = independent variable, % water; X_2_ = independent variable, l:s ratio.

**Table 2:** HPLC-DAD-MS analysis of (poly)phenol-enriched extracts (PEEs) from raw and thermally treated *Ribes magellanicum* fruits, extracted with EtOH:H_2_O:CH_3_COOH (75:24:1) and a solvent-to-solid ratio of 20 mL/g. Compounds were detected by means of UV detection set at 254 nm and tentatively identified by mass spectrometry. Compounds 1A to 13A and 1B to 15B correspond to PEEs from raw and thermally treated samples, respectively.

Peak	Retention Time (min)	λmax (nm)	F. Weight (g/mol)	Ion Detection Mode (+ or −)	Detected Mass (Fragmentation) (m/z)	Tentative Identification
1A	5.65	322, 214	354	−	353 (191, 179, 135)	Caffeoyl quinic acid
2A	7.37	320, 260	354	−	353 (191, 179, 135)	Caffeoyl quinic acid
3A	8.27	320, 260	354	−	191, 179, 135	3-Caffeoyl quinic acid
4A	9.61	324, 290 (sh), 240, 222 (sh)	354 180	+ −	355 (285, 268.98) 377 (355) 179 (135)	3-Caffeoyl quinic acid 3-Caffeoyl quinic acid + Na^+^ Caffeic acid
5A	9.95	325, 2920 (sh), 242	342	−	341 (161, 133)	Caffeoyl hexoside
6A	14.24	340, 280, 240	368	+	369 (207, 185)	Feruloylquinic acid
7A	16.17	520, 280, 240	448 449	− +	447 (285, 255) 450 (287)	Kaempferol hexoside Cyanidin glucoside
8A	17.79	354, 266, 244	448	+	460 (286)	Kaempferol derivate
9A	18.23	354, 254	464 154	− −	463 (301, 271, 255) 153 (125, 107, 83)	Quercetin hexoside Phloroglucynaldehyde
10A	20.05	354, 246	464	+	466 (303)	Quercetine hexoside
11A	20.92	350, 266, 246	449	−	447 (285)	Kaempferol derivate
12A	21.52			−	447 (285)	Kaempferol derivate
13A	23.36			−	447 (285)	Kaempferol derivate
1B	5.57	322, 306 (sh), 220	354	−	353 (328, 229, 191, 179, 135)	Caffeoyl quinic acid derivative
2B	7.29	320, 260 314, 398, 240	354 154	− −	353 (191, 179, 135) 153 (109)	Caffeoyl quinic acid Protocatechuic acid
3B	8.2	320, 240	336	−	335 (161, 133)	Dehydrated 3-caffeoyl quinic acid
4B	9.54	324, 290 (sh), 240, 220 (sh)	354	−	353 (191, 179, 135, 134) 375 (353)	3-Caffeoyl quinic acid
5B	11.30	310, 236	350	−	349 (328, 187, 178, 164)	Caffeoyl quinic acid oxidized derivative
6B	16.35	520, 280, 240	594	−	593 (447, 343, 285) 595 (449, 355, 287)	Cyanidin hexoside rahmnoside
7B	17.73	350, 268 (sh), 240			447 (285)	Kaempferol hexoside
8B	18.2	344, 266	620 154	− −	619 (317) 153 (125, 107, 83)	Petunidin derivative Phloroglucynaldehyde
9B	19.85	352, 266 (sh), 240	610	−	609 (301, 271, 255, 243,151)	Rutin
10B	20.88	348, 266, 244	464 432	− −	463 (382, 343, 301, 254) 431 (269)	Quercetin hexoside Apigenin hexoside
11B	21.35		594	−	593 (363, 336, 284-284, 255) 561 (460, 350) 784 (460, 360, 294) 789 (478, 407, 350, 241) 696 (460, 246) 617 (460, 331) 460 (301)	Kaempferol 3-o-rutinoside Quercetin hexoside
12B	23.33	348, 268, 242	490	−	489 (285)	Kaempferol hexoside monoacetate
13B	24.84	348, 268, 242	464	−	447 (301)	Quercetin hexoside
14B	26.82	344, 268, 242	432	−	431 (285, 133)	Kaempferol rhamnoside
15B	30.77	276, 234	348	−	347 (303, 233)	Quercetin oxidative derivate

**Table 3:** Factorial design of experiments (DOE) for the evaluation of the contribution of factors to the activity and yield of extracts.

Experiment	Factorial DOE		Description of Variables	
Number	% Water	l:s Ratio	X_1_ % Water	X_2_ l:s Ratio
1	−1	−1	0	5 mL/g
2	−1	0	0	10 mL/g
3	−1	1	0	20 mL/g
4	0	−1	24	5 mL/g
5	0	0	24	10 mL/g
6	0	1	24	20 mL/g
7	1	−1	49	5 mL/g
8	1	0	49	10 mL/g
9	1	1	49	20 mL/g

## Data Availability

Data are contained within the article and Supplementary Materials.

## Supplementary Materials

The following supporting information can be downloaded at https://www.mdpi.com/article/10.3390/molecules30020318/s1, Table S1: Changes on extraction yield, cyanidin 3-glucoside amounts and antioxidant activity of raw and thermally treated (TT) *Ribes magellanicum* fruits extracted at different solvent-to-solid ratios with three different solvent mixtures (ethanol: acetic acid (99:1), ethanol: water: acetic acid (75:24:1) and ethanol: water: acetic acid (50:49:1); Table S2: Changes on the half maximal inhibitory concentration (IC50) of oxidation products (NFK, Di-Tyr and Kyn) and advanced glycation end products (AGEs1 and AGEs2) generated by the incubation of bovine serum albumin with glucose, when incubated with polyphenolic enriched extracts (PEEs) from raw and thermally treated (TT) *Ribes magellanicum* fruits. PEEs were extracted at different solvent-to-solid ratios with three different solvent mixtures (ethanol: acetic acid (99:1), ethanol: water: acetic acid (75:24:1) and ethanol: water: acetic acid (50:49:1).
