# Supplementary material for: Effect of Thermal Treatment on the Extraction and Antioxidant and Antiglycation Activities of (Poly)phenols from Ribes magellanicum
Source: Molecules. 2025 Jan 15;30(2):318. doi: 10.3390/molecules30020318 (PMC11767453; doi:10.3390/molecules30020318)
Supplement: Supplementary file 1 [file molecules-30-00318-s001.zip › molecules-3352528-supplementary.pdf]

Table S1

Changes on extraction yield, cyanidin 3-glucoside amounts and antioxidant activity of raw and thermally treated (TT) *Ribes magellanicum* fruits extracted at different solvent-to-solid ratios with three different solvent mixtures (ethanol: acetic acid (99:1), ethanol: water: acetic acid (75:24:1) and ethanol: water: acetic acid (50:49:1).

Number	Sample code	Solvent and treatment	Solvent to solid ratio (mL/g)	Extraction yield (%)*	Total polyphenols (mg GAE/g PEE)	ORAC (µmol TE/L)	TEAC (µM TE /g PEE)	Cyanidin 3-glucoside (µmol/g PEE)
1	A	EtOH:H_2O (99:1)/raw	5	1.01 ± 0.15	164.39 ± 4.96	2654.37 ± 69.65	2054.2 ± 75.4	28.9 ± 0.3
2	AC	EtOH:H_2O (99:1)/TT	5	1.14 ± 0.14	89.14 ± 5.72	2337.97 ± 84.05	1924.7 ± 66.1	23.8 ± 10.8
3	B	EtOH:H_2O (99:1)/raw	10	0.84 ± 0.05	156.19 ± 11.69	2409.90 ± 109.79	3146.2 ± 131.8	39.8 ± 21.5
4	BC	EtOH:H_2O (99:1)/TT	10	1.51 ± 0.43	116.76 ± 3.97	3787.83 ± 214.99	1814.3 ± 80.5	39.2 ± 9.2
5	D	EtOH:H_2O (99:1)/raw	20	0.87 ± 0.12	170.53 ± 9.87	2928.37 ± 148.34	3109.5 ± 116.1	48.1 ± 15.3
6	DC	EtOH:H_2O (99:1)/TT	20	1.17 ± 0.17	116.62 ± 5.68	3943.69 ± 140.87	1945.6 ± 75.7	22.3 ± 15.0
7	E	EtOH:H_2O:CH_3COOH (75:24:1)/raw	5	4.52 ± 0.23	211.40 ± 2.53	2854.03 ± 62.71	3075.6 ± 135.1	40.6 ± 16.9
8	EC	EtOH:H_2O:CH_3COOH (75:24:1)/TT	5	3.54 ± 0.19	169.47 ± 3.18	2138.53 ± 46.91	3519.7 ± 160.7	14.7 ± 4.1
9	F	EtOH:H_2O:CH_3COOH (75:24:1)/raw	10	4.58 ± 0.28	189.87 ± 4.29	2367.49 ± 18.62	3616.9 ± 53.7	86.2 ± 73
10	FC	EtOH:H_2O:CH_3COOH (75:24:1)/TT	10	4.13 ± 0.29	167.35 ± 7.53	2225.97 ± 18.06	3746.2 ± 52.8	14.1 ± 3.5
11	G	EtOH:H_2O:CH_3COOH (75:24:1)/raw	20	4.36 ± 0.21	205.86 ± 3.89	1950.90 ± 42.99	3729.5 ± 53.3	61.4 ± 15.2
12	GC	EtOH:H_2O:CH_3COOH (75:24:1)/TT	20	4.19 ± 0.13	225.69 ± 5.28	2025.64 ± 15.33	3418.7 ± 48.9	10.5 ± 2.2
13	H	EtOH:H_2O:CH_3COOH (50:49:1)/raw	5	4.58 ± 0.2	213.14 ± 5.27	3255.63 ± 54.92	3238.5 ± 47.1	62.1 ± 8.7
14	HC	EtOH:H_2O:CH_3COOH (50:49:1)/TT	5	3.56 ± 0.18	217.79 ± 3.09	2086.77 ± 31.05	3311.7 ± 54.2	20.2 ± 4.9
15	I	EtOH:H_2O:CH_3COOH (50:49:1)/raw	10	6.04 ± 0.56	214.60 ± 4.29	2160.53 ± 12.95	3737.7 ± 65.8	44.7 ± 8.6
16	IC	EtOH:H_2O:CH_3COOH (50:49:1)/TT	10	3.69 ± 0.1	184.48 ± 1.61	2302.23 ± 44.30	3241.5 ± 54	14.7 ± 3.2

17	J	EtOH:H_2O:CH_3COOH (50:49:1)/raw	20	6.63 ± 0.58	217.75 ± 4.35	2537.40 ± 55.36	4079.1 ± 61.3	35.3 ± 7.5
18	JC	EtOH:H_2O:CH_3COOH (50:49:1)/TT	20	3.75 ± 0.41	164.95 ± 1.87	2584.73 ± 26.14	2892.9 ± 47.9	2.3 ± 0.9

*Calculated based on dry mass, TE: Trolox equivalents, GAE: gallic acid equivalents.

Table S2

Changes on the half maximal inhibitory concentration (IC_{50}) of oxidation products (NFK, Di-Tyr and Kyn) and advanced glycation end products (AGEs1 and AGEs2) generated by the incubation of bovine serum albumin with glucose, when incubated with polyphenolic enriched extracts (PEEs) from raw and thermally treated (TT) *Ribes magellanicum* fruits. PEEs were extracted at different solvent-to-solid ratios with three different solvent mixtures (ethanol: acetic acid (99:1), ethanol: water: acetic acid (75:24:1) and ethanol: water: acetic acid (50:49:1).

Number	Sample code	Solvent and treatment	Solvent to solid ratio (mL/g)	IC_{50} NFK (µg/mL)	IC_{50} AGEs1 (µg/mL)	IC_{50} Di-Tyr (µg/mL)	IC_{50} Kyn (µg/mL)	IC_{50} AGEs2 (µg/mL)
1	A	EtOH:H_2O (99:1)/raw	5	14.20 ± 2.45	14.86 ± 2.58	14.39 ± 2.27	42.12 ± 4.94	51.56 ± 5.15
2	AC	EtOH:H_2O (99:1)/TT	5	21.48 ± 1.86	21.93 ± 1.75	21.67 ± 1.72	65.73 ± 11.87	73.25 ± 2.37
3	B	EtOH:H_2O (99:1)/raw	10	33.66 ± 0.23	32.62 ± 2.31	32.51 ± 2.16	59.68 ± 6.69	85.01 ± 6.55
4	BC	EtOH:H_2O (99:1)/TT	10	20.64 ± 1.29	20.87 ± 1.25	21.14 ± 1.27	40.02 ± 1.1	48.93 ± 3.8
5	D	EtOH:H_2O (99:1)/raw	20	45.78 ± 1.89	48.72 ± 4.22	45.71 ± 3.02	141.42 ± 25.65	118.91 ± 1.67
6	DC	EtOH:H_2O (99:1)/TT	20	26.35 ± 2.41	26.55 ± 2.08	27.03 ± 2.27	85.32 ± 13.38	78.45 ± 6.56
7	E	EtOH:H_2O:CH_3COOH (75:24:1)/raw	5	24.83±2.9	24.85±1.5	24.40 ± 1.03	67.05 ± 3.09	76.94 ± 11.5
8	EC	EtOH:H_2O:CH_3COOH (75:24:1)/TT	5	21.74 ± 1.4	21.76 ± 1.36	22.47 ± 1.13	79.56 ± 3.03	90.81 ± 6.68
9	F	EtOH:H_2O:CH_3COOH (75:24:1)/raw	10	22.63 ± 1.1	22.84 ± 1.31	23.32 ± 3.12	76.39 ± 8.13	76.19 ± 4.3
10	FC	EtOH:H_2O:CH_3COOH (75:24:1)/TT	10	17.23 ± 0.45	17.81 ± 0.67	11.06 ± 1.22	48.08 ± 4.64	57.15 ± 3.46
11	G	EtOH:H_2O:CH_3COOH (75:24:1)/raw	20	24.90 ± 0.47	24.76 ± 0.3	25.45 ± 0.36	36.45 ± 0.83	28.28 ± 0.66
12	GC	EtOH:H_2O:CH_3COOH (75:24:1)/TT	20	24.05 ± 0.5	23.97 ± 0.53	24.71 ± 0.53	32.85 ± 2.42	25.48 ± 2.22
13	H	EtOH:H_2O:CH_3COOH (50:49:1)/raw	5	28.33 ± 0.86	28.18 ± 0.71	28.78 ± 0.46	49.78 ± 2.34	36.44 ± 1.53

14	HC	EtOH:H_2O:CH_3COOH (50:49:1)/TT	5	38.29 ± 0.1	37.63 ± 0.06	40.74 ± 0.41	42.19 ± 0.23	31.44 ± 0.37
15	I	EtOH:H_2O:CH_3COOH (50:49:1)/raw	10	26.98 ± 0.27	26.98 ± 0.24	27.97 ± 0.36	44.09 ± 0.73	32.96 ± 0.78
16	IC	EtOH:H_2O:CH_3COOH (50:49:1)/TT	10	48.63 ± 1.24	48.27 ± 1.21	51.63 ± 1.16	63.34 ± 3.03	52.56 ± 3.75
17	J	EtOH:H_2O:CH_3COOH (50:49:1)/raw	20	43.24 ± 1.28	42.87 ± 1.11	44.69 ± 0.94	51.42 ± 1.2	39.78 ± 0.91
18	JC	EtOH:H_2O:CH_3COOH (50:49:1)/TT	20	39.93 ± 1.99	39.39 ± 1.91	41.77 ± 1.75	48.75 ± 3.56	41.55 ± 4.06
